# The Unveiled Potential of Telehealth Practice in Vestibular Rehabilitation: A Comparative Randomized Study

**DOI:** 10.3390/jcm13237015

**Published:** 2024-11-21

**Authors:** Andreas Lilios, Christos Nikitas, Charalampos Skoulakis, Aggeliki Alagianni, Ioannis Chatziioannou, Panagiota Asimakopoulou, Theognosia Chimona

**Affiliations:** 1 Department of Otolaryngology, Head and Neck Surgery, University Hospital of Larisa, 413 34 Larisa, Greece; andreaslilios@yahoo.gr (A.L.); jhajiioannou@gmail.com (I.C.); 2 1st Department of Otolaryngology, Head and Neck Surgery, National and Kapodistrian University of Athens, Hippocrateion General Hospital, 115 27 Athens, Greece; xnikitas@hotmail.com; 3 Department of Otolaryngology, Head and Neck Surgery, Chania General Hospital, 733 00 Chania, Greece

**Keywords:** chronic vertigo, chronic unilateral vestibular hypofunction, vestibular rehabilitation, telehealth practice

## Abstract

**Background and Objectives:** Unilateral vestibular hypofunction (UVH) in cases of insufficient central nervous system compensation leads to chronic dizziness. A customized vestibular rehabilitation (VR) program is more beneficial than a generic set of exercises for patients with chronic UVH. The purpose of the present study is to compare a customized remotely supervised VR program with a customized unsupervised VR program in chronic UVH patients. **Methods:** Participants were randomly allocated into two groups (Group A and Group B) and received an individualized 6-week home-based VR program that included adaptation and habituation exercises, balance and gait training. All individuals’ VR program implementation was evaluated weekly, allowing for exercise modifications. Moreover, Group A received additional remote supervision via phone communication (twice per week). The effectiveness of each VR program was evaluated using the scores of the Mini-BESTest, the Functional Gait Assessment (FGA), and the Dizziness Handicap Inventory (DHI). **Results:** At the 6-week assessment, participants in Group A had significantly better scores in objective and subjective evaluated parameters than those in Group B (*p* < 0.001). Group A also showed better compliance with the VR program. On the contrary, participants’ conformity in their individualized exercises was not affected by gender, duration of symptoms, or BMI (*p* > 0.05). **Conclusions:** Our clinical study highlights the advantages of using telephone communication, with a structured design and implementation, to assist individuals in successfully following a VR protocol.

## 1. Introduction

Dizziness is among the most frequent complaints reported by individuals visiting emergency departments, with vestibular neuritis (VN) being the second leading etiology of peripheral vestibular vertigo [1]. VN causes unilateral vestibular hypofunction (UVH) and affects both genders equally, with the highest incidence occurring between 40 and 50 years of age [2,3]. Multiple theories have been implicated in its pathophysiology, such as neurotropic viral infections or reactivations, vascular disorders, autoimmune diseases, and allergies [1,2,4].

An acute UVH episode presents with vertigo, nausea, emesis, and imbalance. These symptoms subside with time through central compensation which is achieved with brain reorganization, leading to recovery and functional rehabilitation [5,6]. UVH results in impairment in both static and dynamic balance functions. The static deficits, encompassing postural and oculomotor issues, typically resolve within a few days. However, the dynamic deficits involve reduced gain and abnormal timing of vestibulo-ocular and vestibulospinal reflexes, which gradually ameliorate over several weeks. However, the final compensation achieved for dynamic deficits is typically more limited than that for static syndromes [5,7,8,9]. In almost one-third of patients with UVH, the central nervous system does not compensate or compensate insufficiently, and these patients experience chronic dizziness [10]. In these cases, patients complain of dizziness, blurred vision, instability in gait and posture, and compromised spatial orientation and navigation [11,12]. Consequently, chronic dizziness adversely affects patients’ quality of life, negatively impacting daily functional activities, work, driving abilities, and socializing [13,14].

Current pharmacological therapy is limited to managing acute symptoms and has no effect in treating the underlying causes of VN or promoting compensation [15,16,17]. Thus, vestibular rehabilitation (VR) is the treatment modality of choice for UVH patients, with numerous studies reporting improvement in dizziness, balance, and overall quality of life [12,13,18,19,20,21,22].

VR programs include habituation, adaptation, and substitution exercises combined with balance and gait exercises [21,23,24]. The habituation exercises are based on the idea that prolonged exposure and repeated movement to a provocative stimulus (e.g., head–body movements) will gradually reduce the motion-provoked symptoms [25]. The adaptation exercises are targeted to the impaired VOR, known as the “VORx1” and “VORx2” exercises. In the VORx1 exercise, the individuals fix their gaze on a stationary target by moving their heads horizontally or vertically. During the VORx2 exercises, both the head and the target move horizontally or vertically in opposite directions while the gaze remains fixed on the target [26]. Finally, the main goal of the substitution exercises is to use alternative strategies for the missing vestibular function. A potential substitution of vestibulospinal reflex involves using proprioceptive, visual cues, or both for static and dynamic balance maintenance. Finally, the cervical ocular reflex, smooth pursuit eye movements, and the central preprogramming of eye movements are used to substitute the reduced gain of VOR [27,28]. Balance exercises under challenging dynamic and sensory conditions are also important to a VR program. Balance exercises reduce visual-provoked symptoms and correct the overdependence on proprioceptive and visual inputs. They should be applied to UVH patients with postural control deficits [14,28,29].

It is well documented in the literature that customized VR exercises are more beneficial than a generic set of exercises for patients with chronic UVH [30,31]. Furthermore, supervised VR programs promote continued participation and adherence; they reduce dropouts and possibly lead to better outcomes than unsupervised VR programs. If unsupervised, mobility dysfunction and cognitive impairment may lead to deficient adherence and limited progress and recovery [13]. The regular evaluation of progress throughout supervised VR enables the therapist to incorporate more demanding balance exercises in a timely manner and ensures the correct execution of the whole program.

Several critical issues still exist, such as the duration of VR programs, the exercise repetitions, and the type of supervision. In the current literature, no clinical studies are investigating the effectiveness of telehealth/remote supervised VR therapy. The purpose of the present study is to compare a customized remotely supervised VR program with a customized unsupervised VR program in chronic UVH patients and to investigate the role of remote VR support on patients’ compliance and adherence.

## 2. Materials and Methods

### 2.1. Study Design

This prospective randomized clinical trial compares the effectiveness of a remotely supervised customized VR program with that of an unsupervised customized VR program in patients with chronic UVH. The study’s inclusion criteria were as follows: (a) age between 18 and 65 years, (b) symptoms duration greater than six weeks, (c) confirmed UVH, i.e., ≥22% unilateral weakness in caloric testing at videonystagmography, and (d) participant’s written informed consent. The exclusion criteria were: (a) CNS disorders (e.g., degenerative diseases, multiple sclerosis), (b) fluctuating symptoms (e.g., Meniere’s disease, benign paroxysmal positional vertigo vestibular migraine), (c) bilateral vestibular hypofunction (both total Right and Left caloric-induced nystagmus slow phase velocity (SPV) < 12 deg/s), (d) chronic orthopedic disorder affecting static and dynamic balance, (f) poor cooperation and communication, (e) treatment with CNS-acting medications (e.g., sedatives, hypnotics), and (g) severe anxiety and depression (Hospital Anxiety and Depression Scale score > 15). The study was conducted in accordance with the Declaration of Helsinki, and approved by the ethics committee of the University Hospital of Larissa (code 7/11, protocol number 27746/14 June 2024).

### 2.2. Patients and Allocation

Fifty envelopes were shuffled with an allocation indication for a specific group (Group A: 25 envelopes, Group B: 25 envelopes). Fifty sequential patients with the diagnosis of UVH who fulfilled the study’s criteria enrolled in the study. The diagnosis of UVH was confirmed after a comprehensive clinical and laboratory evaluation at the Audiology-Neurotology-Laboratory of the ENT Department in the University Hospital of Larisa (Larisa, Greece). Participants were randomly allocated to the two groups by the otolaryngologist who performed the clinical and laboratory evaluation. Each time a patient was found suitable for the study, a sealed envelope was opened, and the patient was assigned to one of the two study groups (Group A = 25 participants, Group B = 25 participants).

All participants were informed about the effectiveness of VR therapy, the therapeutic intervention, and the program duration (total intervention time, daily exercise frequency, and duration of each exercise separately). Written informed consent was obtained from each participant, who had the right to withdraw from the study at any time.

### 2.3. Intervention

Patients’ demographic data and history, including gender, age, body mass index (BMI), educational level, occupation, dizziness-related symptoms, comorbidities, and medication intake, were recorded.

Participants in each group (Group A and Group B) received an individualized six-week home-based VR program that included adaptation and habituation exercises as well as balance and gait training. A specialized physical therapist performed a clinical assessment at the time of enrollment (baseline assessment) and at the end of the sixth week.

All individuals (Group A and Group B) were asked to visit the Audiology-Neurootology Laboratory weekly to evaluate the VR program implementation. Depending on their condition and reported symptoms during the VR exercises, their program underwent modifications that either facilitated or progressed the exercises in difficulty. Moreover, Group A received additional remote supervision via phone communication (twice per week), allowing for exercise modifications as necessary, according to each patient’s clinical condition, progress, and individual needs. Thus, modifications of the VR program in Group A were also based on 10 questions that were asked via a telephone call on a pre-arranged appointment (Table 1).

The adaptation and habituation exercises prescribed to the patients in both groups were customized to last 30 min daily. Additionally, the frequency of the exercises was set at 3 times per day (10 min per session/3 times per day). Adaptation exercises included VORX1 and VORX2 to enhance VOR gain. Habituation exercises were prescribed according to head or body movements that trigger symptoms following the Motion Sensitivity Test. Additionally, all patients received a customized balance and gait exercise program.

The progression of adaptation exercises included increasing the velocity of head movements, extending the duration (1–2 min), changing positions (sitting—standing—walking), changing the target’s distance (near to far), and changing target placement in front of a distracting visual pattern (a chessboard paper panel 1.5 × 2 m was provided to each patient). The progression of habituation exercises included changes of posture (e.g., exercise performance from sitting position to standing), longer exercise duration (e.g., 1–2 min), greater range of motion, and change of movement velocity (from slow to faster). The progression of balance exercises included modifications of the support base from stable to unstable, variations in visual inputs and changes in the support base, and finally, gait exercises progression included changes in direction, walking with speed variations, walking on uneven surfaces, and head movements during walking, in sagittal, frontal, and diagonal planes.

Participants were handed a table-format week diary and encouraged to record the frequency of the individualized exercises performed and any discomfort they experienced during their VR program for each day. They were also advised to contact the physical therapist via phone or email with any questions or problems they faced with their exercises.

### 2.4. Evaluation

Balance, gait performance, and subjective symptoms were evaluated using the scores of the Mini-BESTest, the Functional Gait Assessment (FGA), and the Dizziness Handicap Inventory (DHI). The physical therapist who developed the individualized VR program evaluated and recorded each participant at baseline and at the end of the sixth week. The number of adaptation-habituation exercise times performed was also estimated, reflecting participants’ compliance with a max of 126 times through the 6-week VR program.

The Mini-BESTest is a 14-item test that assesses reactive postural responses, anticipatory postural adjustments, sensory orientation, and dynamic gait. Each item is scored from 0 to 2 (0 = severe performance impairment; 1 = moderate performance impairment; 2 = normal performance) on a 3-level ordinal scale, with a maximum score of 28 points. Moreover, the minimal detectable change (MDC) is from 3.8 to 3.5 points with a minimum clinically important difference of 4 points [32,33,34].

FGA comprises 10-item high-equilibrium risk trials and assesses multiple motor tasks during walking and postural stability [35]. Each item is scored from 0 to 3 (0 = severe impairment, 3 = normal) on an ordinal scale, with lower scores indicating more significant disability and a maximum total score of 30 points. The FGA’s MDC is 4 points [36].

The Dizziness Handicap Inventory (DHI) questionnaire quantifies the subjective perception of symptoms and their impact on daily functional activities in patients with vestibular disorders [37,38]. It is a 25-item self-assessment questionnaire focusing on dizziness’s effect on three domains: physical, emotional, and functional. Three alternative responses can be given (yes = 4, sometimes = 2, no = 0), and total values range from 0 to 100. The higher the score, the more significant the disability (i.e., 0–30 = mild disability, 31–60 = moderate disability, 61–100 = severe disability) with a minimum clinically important difference of 18 points [38].

### 2.5. Statistical Analysis

Summary descriptive statistics are presented as mean (standard deviation, SD) for continuous variables and frequency (%) for categorical variables. Changes in the evaluated parameters within the same group were assessed using the Bonferroni test. Baseline assessment results for the two groups and treatment outcomes were compared using *t*-test with the level for statistical significance set at *p* < 0.005. Results with *p*-values between 0.05 and 0.005 were considered “suggestive”. All statistical analyses were performed with SPSS (Statistical Package for Social Sciences (SPSS), version 26.0, Chicago, IL, USA).

## 3. Results

Table 2 shows the groups’ general characteristics at enrollment. The mean age of participants in groups A and B was 50.9 years and 49.5 years, respectively (*p* = 0.73). The mean duration of symptoms in Group A was 14.3 weeks, while in Group B, it was 12.8 weeks (*p* = 0.68) (Table 2).

**Figure 1:**
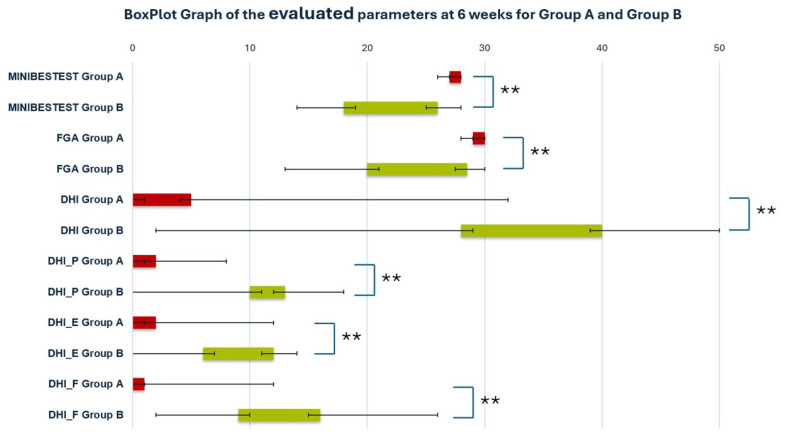
Boxplot graph of the evaluated parameters at 6 weeks for Group A and Group B. ** Statistically significant difference. Red boxes: Group A, green boxes: Group B.

The initial assessment of the evaluated parameters did not reveal statistically significant differences between the groups (*p* > 0.05), reflecting the homogeneity of the participants after allocation (Table 3). Participants in Group A showed significant improvement in balance control evaluated by the physical therapist (FGA and Mini-BESTest) (*p* < 0.001) at the 6-week evaluation. In contrast, Group B showed a suggestive significant improvement with a *p*-value of 0.0052 and 0.02, respectively (Table 3). The self-assessment symptoms’ perception, using DHI total scores and its subcategories, was significantly improved at the end of the study for both groups (*p* < 0.001) (Table 3). At the 6-week assessment, participants in Group A had significantly better scores in objective and subjective evaluated parameters than those in Group B (*p* < 0.001) (Table 3, Figure 1). Post hoc power analysis revealed a power 87% at the 5% alpha level, for all evaluated parameters, with a large effect size of 0.8.

**Figure 2:**
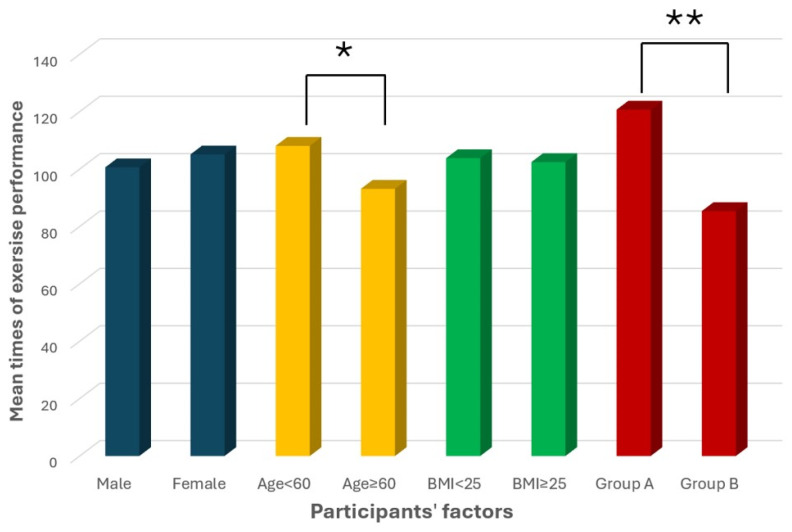
Mean times of exercise performance vs. participants’ factors. * Suggestive of statistical difference. ** Statistically significant difference.

Our study population showed that compliance with the VR program was significantly better in Group A, which received additional remote supervision twice a week, with a mean exercise performance of 120.7 times. The mean phone calls’ duration was 8.4 min (SD 1.8). On the contrary, participants’ conformity in their individualized exercises was not affected by gender, duration of symptoms, or BMI (*p* > 0.05). Patients under 60 showed suggestive better compliance than older patients (*p* = 0.012) (Table 4 and Figure 2).

## 4. Discussion

Our prospective randomized clinical trial investigated the effect of telerehabilitation on disability level and posture in individuals with chronic UVH. The diagnosis of chronic UVH was based on caloric stimulation results during videonystagmography. The cut-off value for the diagnosis of UVH varies among laboratories from 20% to 30%, with a value of 25% being the most common [39]. We preferred to use a lower cut-off value (i.e., 22%, which is routinely used in our labs), as we could include symptomatic patients with milder asymmetry. It appears that individuals who received additional remote supervision by telephone (Group A) had near-perfect compliance with the treatment protocol compared to the control group (Group B), which seems to have lost about 13 days of exercise, on average, compared to the total protocol duration (~42 days). This is the main factor whereby although the results showed an in-group improvement in all outcome measures for both study groups, there was also a statistically significant between-group difference both in terms of functional gait and postural control (FGA and Mini-BESTest, respectively), but also for the level of disability (DHI) where the difference even at its lowest value overlaps the minimal clinically significant difference. A remarkable strength point of the current study is that the statistical analysis was performed with a “statistically significant” level set at *p* < 0.005. Furthermore, the participants in Group A, through a non-time-consuming remote assessment, were able to ensure that the exercises were performed promptly and to increase the difficulty of the exercise program more quickly than Group B. These two factors (better compliance and rapid progression) most likely contributed to the statistically significant difference observed between the groups both in terms of functional gait and postural control (FGA and Mini-BESTest, respectively), but also for disability level (DHI), where the difference even at its lowest value overlapped the minimal clinically significant difference.

The improvement observed in both study groups confirms the robust literature summarized in the revised clinical guidelines on the efficacy of VR in people with chronic UVH [13]. The same clinical guidelines provide strong evidence that a supervised rehabilitation program improves adherence and outcomes. Although these guidelines recommend weekly clinic visits, they do not mention the optimal dosage of supervision nor any advantage in delivering modalities and urge researchers to investigate this. In a recent systematic review, although all studies of supervised vestibular rehabilitation showed better clinical outcomes, overall, they did not provide strong evidence as to the superiority of supervision [19].

It is known that the stronger the therapeutic relationship between patient and health professional, the better the therapeutic outcome. This is well documented in several pathologies, especially in people with symptoms of dizziness [40,41,42]. Highly person-centered telephone sessions offer encouragement and reassurance to people with dizziness, building a strong relationship [43]. This approach allows the patient to feel safe and to modify their perceptions. Considering the strong interconnection of vestibular and limbic neural circuits and the high-stress levels seen in this population, we perceive that the opportunity for individuals in the intervention group to provide immediate feedback and have ongoing support was beneficial [44,45]. Empathy is one of the most critical factors in promoting an excellent patient–therapist relationship, and the development of good communication skills to enhance this and establish a patient-oriented approach is prioritized in the education of physiotherapists [46].

Of particular interest is the fact that enhancing the therapeutic relationship through telephone communication improved the subjective perception of patients’ symptoms and their postural control. We know that perception of self-stability is linked to the limbic system function and can be modulated by expectations [47]. Functional dizzy patients experience a highly conscious control over locomotion as their threat-assessment cortical center, which arises from their limbic system, alters sensory input weighting, leading to persistent perceptual distortions [48]. By reducing threat levels through frequent communication, which leads to more frequent assessment of symptoms, modification of the treatment protocol, and thus enhancing self-confidence and providing reassurance, participants manage to abandon the hyper-vigilance postural model that is biased and adopt efficient executive postural control.

Using a mobile phone for patient–therapist communication to facilitate better supervision of a therapeutic protocol is the simplest and most widespread form of telerehabilitation. It requires no special equipment since mobile phones are an integral part of modern social life. They are easy to use, have easy set-up and usable functionality, are convenient, and allow real-time interaction at a basic level. It also prevents unnecessary hospital visits, maintaining a high level of continuity of care. Despite so many apparent advantages, its widespread application is under-researched in terms of clinical benefits and comparison with other forms of telehealth. A recent systematic review showed no differences in treatment effectiveness, patient satisfaction, and cost-effectiveness between telephone and video telehealth consultations, considering both options equally safe and effective [49]. Nevertheless, the included studies were not recent. They showed many methodological issues (inadequate sample power, concerns about the randomization procedure, and deviations from the intended intervention), and their conclusions cannot be generalized [49]. On the other hand, a recent meta-analysis demonstrated that telerehabilitation modalities for dizziness offer a significant clinical advantage over standard care concerning disability, anxiety, and dizziness frequency and severity [50].

Our clinical study highlights the advantages of applying telephone communication, with a structured design and implementation, to individuals following a vestibular rehabilitation protocol. We recommend that clinicians involved in vestibular rehabilitation follow the modified questionnaire in Table 1 and incorporate it directly into their clinical practice. Telehealth is a new area in medicine regarding diagnosis and treatment, and of course, as technology advances, this area will also evolve. However, a very high percentage of physiotherapists (95.5%) with experience in vestibular rehabilitation do not integrate telerehabilitation into their clinical practice, even though this category of adherence strategy has proven to be one of the most effective [51,52].

The limitations of our study include the single-center design, the lack of a control group receiving an in-person weekly VR supervision session, and the use of non-objective measures as VR outcomes. Furthermore, a post hoc power analysis was performed which depends on the existing sample and observations of the particular study and may affect the generalizability of the results.

Whatever future supervision applications are, it is guaranteed that they will be integrated into phones. It is noticeable that during the pandemic, which led to a statistically significant increase in cases with peripheral vestibular pathology symptoms, telerehabilitation showed its advantages in terms of remote monitoring, resources allocation, and continuously personalized interventions [53,54].

It is already evident that the use of the telephone as a practice tool during the physiotherapy session has been accepted by the general population [55]. The average age of the participants in the above study was 39.83 (SD 15.15 years). Thus, it was not shown how older people would have responded. In our research, it appeared that age was a factor that influenced compliance, with individuals over 60 years of age not complying the same as younger individuals.

Future research studies should incorporate objective measures or examine older people’s acceptance of technology, even in its simplest and most widespread form, by using validated questionnaires that already exist for this reason [56]. In addition, they should examine the level of satisfaction of health professionals in terms of cost-effectiveness. Finally, they should also examine their results in several common peripheral vestibular pathologies, exploring similarities and differences in patient profiling, effectiveness, and acceptability.

## 5. Conclusions

Implementing structured and well-designed telephone communication (×2 times per week) and weekly clinic visits in a vestibular rehabilitation intervention enhances compliance and further improves symptom perception, postural control, and gait.

## Figures and Tables

**Table 1:** Phone call communication questions for Group A.

Questions for Phone Call Communication
Do the exercises with the target increase your symptoms? If so, which specific ones?
Do the exercises without the target increase your symptoms? If so, which specific ones?
Do you perform the exercises three times per day for 10 min each time, as instructed?
Do the balance and walking exercises cause you any discomfort? If so, which specific ones?
Do you perform balance and walking exercises for at least 30 min per day as instructed?
Do you perform the exercises at the instructed intensity level?
Does the intensity of the symptoms remain the same? If not, has it been increased or decreased?
Do you wait a while between exercises (e.g., in cases where you experience intense dizziness), or do you proceed directly to the next exercise?
Do you encounter any difficulty in your daily functional activities?
Do you have any questions regarding the instructions you received during your visit to the clinic?

**Table 2:** Groups’ general characteristics at enrollment.

Characteristics	Group A	Group B	*p*-Value
N	25	25	
Females/males	15/10	12/13	
Age years, mean, (SD)	50.96 (2.36)	49.56 (3.29)	0.73
BMI mean, (SD)	27.3 (4.0)	28.7 (7.2)	0.4
Symptom’s duration weeks, mean, (SD)	14.32 (2.53)	12.88 (2.37)	0.68

**Table 3:** Comparison of the evaluated parameters between and within groups.

Comparison of the Evaluated Parameters Between Group A and Group B			
		Mean Difference (95%CI)	*p*-Value
**Initial Evaluation**	Mini-BESTest	−1.32 (−3.8 to 1.21)	0.3
	FGA	−2.7 (−6.1 to 0.6)	0.1
	DHI Total	−2.0 (−13.2 to 9.2)	0.7
	DHI Physical	−2.7 (−5.9 to 0.55)	0.1
	DHI Emotional	−1.04 (−5.0 to 2.92)	0.6
	DHI Functional	2.16 (−4.14 to 8.46)	0.5
**6-Week Evaluation**	Mini-BESTest	4.8 (3.08 to 6.51)	<0.001 **
	FGA	5.4 (3.34 to 7.46)	<0.001 **
	DHI Total	−28.08 (−33.8 to −22.3)	<0.001 **
	DHI Physical	−9.5 (−11.4 to −7.5)	<0.001 **
	DHI Emotional	−6.8 (−8.8 to −4.9)	<0.001 **
	DHI Functional	−11.6 (−14.4 to −8.9)	<0.001 **
**Comparison of the Evaluated Parameters Within Groups (Initial vs. 6-Week Evaluation)**			
		**Mean Difference (95%CI)**	***p*** **-Value**
**Group A**	Mini-BESTest	−10.2 (−12.12 to −8.3)	<0.001 **
	FGA	−11.5 (−13.8 to −9.3)	<0.001 **
	DHI Total	45.2 (37.3 to 53.2)	<0.001 **
	DHI Physical	12.08 (10.3 to 13.8)	<0.001 **
	DHI Emotional	12.9 (10.3 to 15.6)	<0.001 **
	DHI Functional	20.2 (14.8 to 25.6)	<0.001 **
**Group B**	Mini-BESTest	−3.8 (−6.4 to −1.2)	0.0052 *
	FGA	−3.96 (−7.26 to −0.65)	0.02 *
	DHI Total	19.2 (9.4 to 28.9)	<0.001 **
	DHI Physical	5.2 (1.8 to 8.6)	0.003 **
	DHI Emotional	7.12 (3.59 to 10.6)	<0.001 **
	DHI Functional	6.4 (2.19 to 10.6)	0.004 **

* Suggestive of statistical difference. ** Statistically significant difference.

**Table 4:** Evaluating factors affecting VR program compliance.

Factor		N (%)	Mean Times of Exercise Performance (SD)	Mean Difference (95%CI)	*p*-Value
**Gender**	Male	23 (46)	100.6 (21.9)	−4.5 (−4.5 to 5.7)	0.44
	Female	27 (54)	105.11 (18.9)		
**Age**	<60 y	33 (66)	108.1 (17.9)	15.03 (3.5 to 26.5)	0.012 *
	≥60 y	17 (34)	93.12 (21.4)		
**BMI**	<25	22 (44)	103.8 (20.2)	1.39 (−10.3 to 13.1)	0.81
	≥25	28 (56)	102.43 (20.6)		
**Group**	Group A	25 (50)	120.7 (5.2)	35.3 (29.8 to 40.8)	<0.001 **
	Group B	25 (50)	85.36 (12.7)		

* Suggestive of statistical difference. ** Statistically significant difference.

## Data Availability

The data presented in this study are available on request from the corresponding author.
